# 2-Chloro­benzene-1,4-diaminium bis­(dihydrogenphosphate)

**DOI:** 10.1107/S1600536812051434

**Published:** 2013-01-04

**Authors:** Mohamed Lahbib Mrad, Matthias Zeller, Mohamed Rzaigui, Cherif Ben Nasr

**Affiliations:** a Laboratoire de Chimie des Matériaux, Faculté des Sciences de Bizerte, 7021 Zarzouna, Tunisia; b Department of Chemistry, Youngstown State University, One University Plaza, Youngstown, Ohio 44555-3663, USA

## Abstract

The asymmetric unit of the title salt, C_6_H_9_ClN_2_^2+^·2H_2_PO_4_^−^, contains two dihydrogenphosphate anions and one 2-chloro­benzene-1,4-diaminium dication. The H_2_PO_4_^−^ anions are inter­connected through strong O—H⋯O hydrogen bonds to form two-dimensional infinite layers parallel to (001). The organic entities are anchored to the inorganic layers through N—H⋯O hydrogen bonds, and through weak C—Cl⋯O halogen bonds [3.159 (2) Å, 140.48 (7)°]. No π–π stacking inter­actions between neighboring aromatic rings or C—H⋯π inter­actions towards them are observed. Minor disorder is observed for the Cl atom and one hy­droxy group [minor-component occupancy = 3.29 (9)%].

## Related literature

For common applications of organic phosphate complexes, see: Masse *et al.* (1993 ▶). For network geometries, see: Rayes *et al.* (2004 ▶); Oueslati *et al.* (2005 ▶). For reference structural data, see: Kaabi *et al.* (2004 ▶); Chtioui & Jouini (2006 ▶). For halogen bonding, see: Metrangolo & Resnati (2001 ▶, 2008 ▶); Politzer *et al.* (2007 ▶). For van der Waals radii, see: Bondi (1964 ▶).
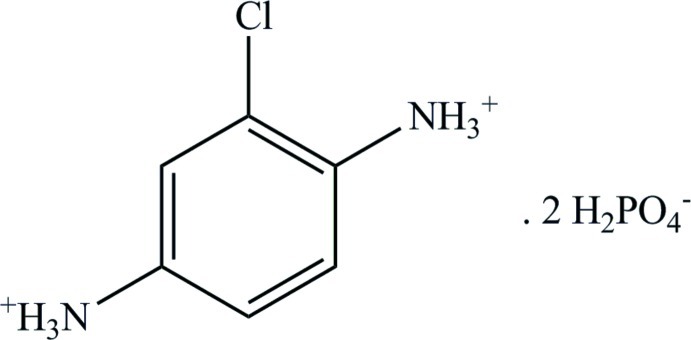


## Experimental

### 

#### Crystal data

C_6_H_9_ClN_2_^2+^·2H_2_PO_4_^−^
*M* *_r_* = 338.57
Orthorhombic, 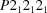

*a* = 7.0084 (8) Å
*b* = 7.9404 (9) Å
*c* = 23.064 (3) Å
*V* = 1283.5 (3) Å^3^
*Z* = 4
Mo *K*α radiation
μ = 0.58 mm^−1^
*T* = 100 K
0.55 × 0.52 × 0.51 mm

#### Data collection

Bruker SMART APEX CCD diffractometer
Absorption correction: multi-scan (*SADABS*; Bruker, 2011 ▶) *T*_min_ = 0.689, *T*_max_ = 0.746
11671 measured reflections
4134 independent reflections
4060 reflections with *I* > 2σ(*I*)
*R*_int_ = 0.021

#### Refinement

*R*[*F* ^2^ > 2σ(*F* ^2^)] = 0.025
*wR*(*F* ^2^) = 0.064
*S* = 1.11
4134 reflections
186 parameters
H-atom parameters constrained
Δρ_max_ = 0.44 e Å^−3^
Δρ_min_ = −0.27 e Å^−3^
Absolute structure: Flack (1983 ▶), 1694 Friedel pairs
Flack parameter: 0.11 (4)

### 

Data collection: *APEX2* (Bruker, 2011 ▶); cell refinement: *SAINT* (Bruker, 2011 ▶); data reduction: *SAINT*; program(s) used to solve structure: *SHELXS97* (Sheldrick, 2008 ▶); program(s) used to refine structure: *SHELXL97* (Sheldrick, 2008 ▶) and *SHELXLE* (Hübschle *et al.*, 2011 ▶); molecular graphics: *SHELXTL* (Sheldrick, 2008 ▶); software used to prepare material for publication: *SHELXTL* and *publCIF* (Westrip, 2010 ▶).

## Supplementary Material

Crystal structure: contains datablock(s) global, I. DOI: 10.1107/S1600536812051434/ru2048sup1.cif

Structure factors: contains datablock(s) I. DOI: 10.1107/S1600536812051434/ru2048Isup2.hkl

Additional supplementary materials:  crystallographic information; 3D view; checkCIF report

## Figures and Tables

**Table 1:** Hydrogen-bond geometry (Å, °)

*D*—H⋯*A*	*D*—H	H⋯*A*	*D*⋯*A*	*D*—H⋯*A*
N1—H1*A*⋯O7^i^	0.91	1.76	2.6726 (15)	175
N1—H1*B*⋯O4^ii^	0.91	1.88	2.7807 (15)	172
N1—H1*C*⋯O2^iii^	0.91	2.05	2.9155 (15)	158
N2—H2*A*⋯O8	0.91	1.88	2.7886 (15)	178
N2—H2*B*⋯O7^iv^	0.91	1.84	2.7450 (15)	178
N2—H2*C*⋯O4	0.91	1.75	2.6545 (15)	175
O1—H1*D*⋯O2^v^	0.84	1.90	2.6525 (14)	148
O3—H3*A*⋯O8^vi^	0.84	1.79	2.5863 (14)	158
O5—H5⋯O8^vii^	0.84	2.00	2.6585 (14)	134
O6—H6*A*⋯O2	0.84	1.79	2.5841 (14)	156
O6*B*—H6*B*⋯O2	0.84	1.84	2.63 (3)	157

Symmetry codes: (i) 
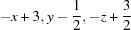
; (ii) 
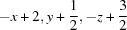
; (iii) 
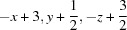
; (iv) 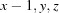; (v) 
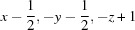
; (vi) 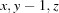; (vii) 
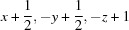
.

**Fig. 1.**
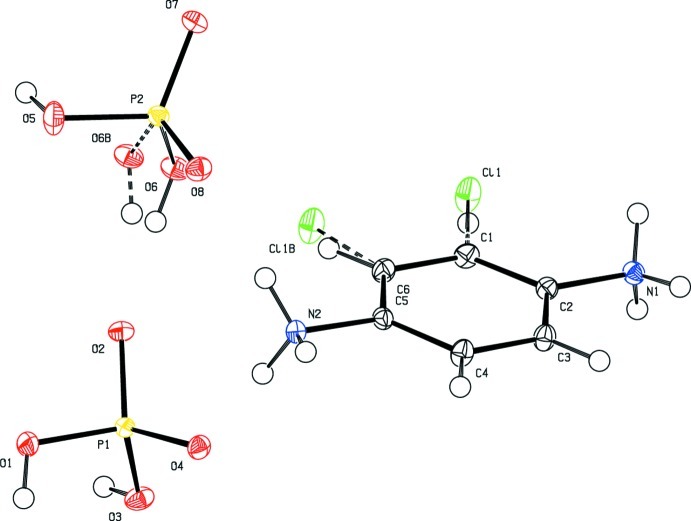
A view of the title compound, showing 50% probability displacement ellipsoids and arbitrary spheres for the H atoms.

**Fig. 2.**
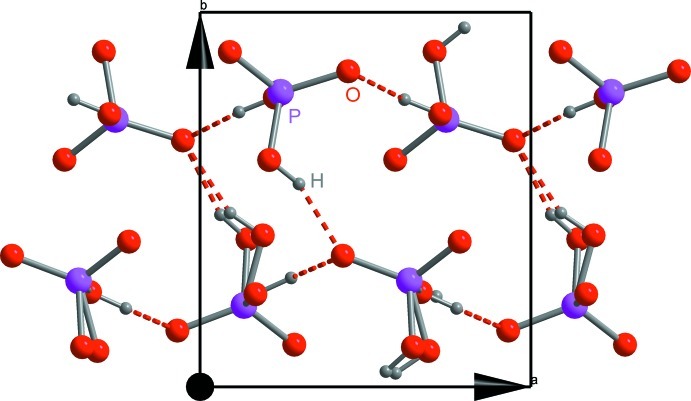
Projection along the c-axis of an inorganic layer in the structure of the title compound. Hydrogen bonds are denoted as red broken lines.

**Fig. 3.**
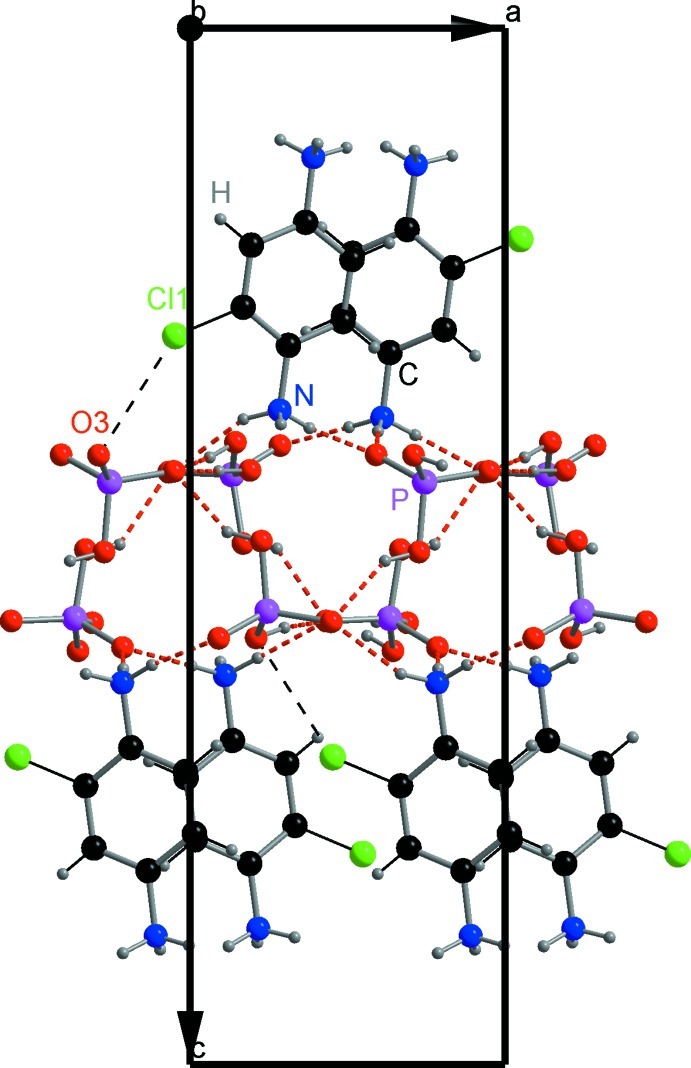
Projection of the structure along the b-axis. Hydrogen bonds are denoted as red broken lines, halogen bonds as black broken lines. For the disordered Cl atom, only the major part is shown.

## supplementary crystallographic information

### Comment

In organic-cation monophosphates, the phosphate anions generally observed are the partially protonated acidic ones H_2_PO_4_^-^ or HPO_4_^2-^. In the solid state such anions are generally interconnected through strong hydrogen bonds so as to build infinite networks with various geometries (Rayes *et al.*, 2004; Oueslati *et al.*, 2005). If these organic-cation monophosphates hybrid materials crystallize in a noncentrosymmetric setting they are of particular interest as nonlinear optical (NLO) materials (Masse *et al.*, 1993). The present work is devoted to the structure of an organic-cation hydrogenphosphate, C_6_H_9_ClN_2_(H_2_PO_4_)_2_, formed by the reaction of 2-chlorobenzene-1,4-diamine with orthophosphoric acid, which crystallized in a non-centrosymmetric setting.

The title organic-inorganic hybrid material, while made from achiral components, crystallizes in the chiral space group *P*2_1_2_1_2_1_. The crystal investigated is partially racemically twinned, with a twinning ratio of 0.89 (4) to 0.11 (4). Its structure consists of one 2-chlorobenzene-1,4-diaminium dication and two crystallographically distinct H_2_PO_4_^-^ anions (Fig. 1). The chlorine atom is disordered over two chemically equivalent positions with a small but noticable presence of the second moiety (refined value 3.29 (9)%). Associated with this disorder is disorder of one phosphate hydroxyl group of one of the H_2_P(2)O_4_^-^ anions, O6. Where not mentioned otherwise, this disorder is ignored in the following more detailed discussion of the structure.

The HPO_4_^2-^ anions show two types of P—O distances depending on whether the oxygen atoms are hydrogen donors or acceptors. As expected, the P—OH distances, varying between 1.54 (3) and 1.581 (1) Å, are significantly longer than the other P—O distances ranging from 1.500 (1) to 1.516 (1) Å. This is in agreement with the literature data (Chtioui & Jouini, 2006; Kaabi *et al.*, 2004). Figure 2 shows that the H_2_PO_4_^-^ anions are interconnected through O—H···O hydrogen bonds to form a two dimensional layer spreading parallel to the (0 0 1) plane at *z* = 0, 1/2 and 1 (Fig. 3). The organic cations, assembled in layers parallel to the H_2_PO_4_^-^ anions at *z* = 1/4 and 3/4, are anchored to the inorganic layers through N—H···O hydrogen bonds whose geometrical characteristics are given in Table 1. The projection of the whole arrangement along the *c*-axis (Fig. 3) shows the alternating cationic and anionic layers. The structure also features a weak C—Cl···O halogen bond between the chlorine atom and one of the H_2_PO_4_^-^ phosphate ions, a type of interaction that has recently attracted high levels of interest due to the observation of such interactions between halogenated compounds and the phosphate moieties in DNA (see *e.g.* Metrangolo & Resnati, 2008). In the title compound the Cl···O distance between Cl1 and O3^i^ is 3.159 (2) Å, the C—Cl···O angle 140.48 (7)° (symmetry operator (i) -*x* + 3, *y* + 1/2, -*z* + 3/2), the equivalent values for the interaction of the minor occupied Cl atom Cl1B with O6B are 2.91 (5) Å and 130 (1)°. While the Cl···O distances are shorter than the sum of the van der Waals radii of chlorine and oxygen (*ca* 3.3 Å, Bondi, 1964), the angles observed are on the small side for C—Cl···O halogen bonds (160–180°, see *e.g.* Politzer *et al.*, 2007; Metrangolo & Resnati, 2001), indicating that the interactions observed are quite weak and more likely a result of the stronger hydrogen bonding interactions rather than one of the major driving forces determining the outcome of the assembly of the structural components of the title compound. No π-π stacking interactions between neighboring aromatic rings or significant C—H···π interactions towards them are observed.

### Experimental

Crystals of the title compound were prepared at room temperature by slow addition of a solution of orthophosphoric acid (6 mmol in 20 ml of water) to an alcoholic solution of 2-chlorobenzene-1,4-diamine (3 mmol in 20 ml of ethanol). The acid was added until the alcoholic solution became turbid. After filtration, the solution was allowed to slowly evaporate at room temperature over several days leading to formation of transparent prismatic crystals with suitable dimensions for single-crystal structural analysis (yield 58%). The crystals are stable for months under normal conditions of temperature and humidity.

### Refinement

The chlorine atom is disordered over two chemically equivalent positions with a small but noticable presence of the second moiety (refined value 3.29 (9)%). Associated with this disorder is disorder of one of the phosphate hydroxyl groups, O6. The minor moiety chlorine and oxygen atoms were constrained to have the same ADPs as their major moiety counterparts. Due to the low prevalence of the minor moiety no disorder was modeled for the aromatic ring the Cl atom is bonded to, despite of the obviously unrealistic C—C—Cl angles for the minor Cl atom.

All non hydrogen atoms were refined anisotropically. All H atoms were located in difference density Fourier maps, but were then placed in calculated positions riding on their respective carrier atom with C—H distances of 0.95, N—H distances of 0.91 Å, and O—H distances of 0.84 Å. Ammonium and hydroxyl H atoms were allowed to rotate but not to tip to best fit the observed electron density distribution. The position of the hydrogen atom of the minor occupied hydroxyl group was refined with a damping factor (DAMP 2000 in *SHELXTL* (Sheldrick, 2008)). In the final refinement cycles after removal of the damping factor its position was set to ride on its carrier oxygen atom. *U*_iso_(H) values were constrained to be 1.2 *U*_eq_(C) of the parent atom for C bound H atoms, and 1.5 times *U*_eq_(N/O) for N and O bound H atoms.

The compound was refined as a racemic twin. The twin ratio refined to 0.89 (4) to 0.11 (4).

### Crystal data

**Table:**

C_6_H_9_ClN_2_^2+^·2H_2_PO_4_^−^	*F*(000) = 696
*M**_r_* = 338.57	*D*_x_ = 1.752 Mg m^−^^3^
Orthorhombic, *P*2_1_2_1_2_1_	Mo *K*α radiation, λ = 0.71073 Å
Hall symbol: P 2ac 2ab	Cell parameters from 7956 reflections
*a* = 7.0084 (8) Å	θ = 2.6–31.8°
*b* = 7.9404 (9) Å	µ = 0.58 mm^−^^1^
*c* = 23.064 (3) Å	*T* = 100 K
*V* = 1283.5 (3) Å^3^	Block, colourless
*Z* = 4	0.55 × 0.52 × 0.51 mm

### Data collection

**Table:**

Bruker SMART APEX CCD diffractometer	4134 independent reflections
Radiation source: fine-focus sealed tube	4060 reflections with *I* > 2σ(*I*)
Graphite monochromator	*R*_int_ = 0.021
ω scans	θ_max_ = 32.0°, θ_min_ = 2.7°
Absorption correction: multi-scan (*SADABS*; Bruker, 2011)	*h* = −6→10
*T*_min_ = 0.689, *T*_max_ = 0.746	*k* = −11→11
11671 measured reflections	*l* = −33→33

### Refinement

**Table:**

Refinement on *F*^2^	Secondary atom site location: difference Fourier map
Least-squares matrix: full	Hydrogen site location: inferred from neighbouring sites
*R*[*F*^2^ > 2σ(*F*^2^)] = 0.025	H-atom parameters constrained
*wR*(*F*^2^) = 0.064	*w* = 1/[σ^2^(*F*_o_^2^) + (0.0328*P*)^2^ + 0.2706*P*] where *P* = (*F*_o_^2^ + 2*F*_c_^2^)/3
*S* = 1.11	(Δ/σ)_max_ = 0.002
4134 reflections	Δρ_max_ = 0.44 e Å^−^^3^
186 parameters	Δρ_min_ = −0.27 e Å^−^^3^
0 restraints	Absolute structure: Flack (1983), 1694 Friedel pairs
Primary atom site location: structure-invariant direct methods	Flack parameter: 0.11 (4)

### Fractional atomic coordinates and isotropic or equivalent isotropic displacement parameters (Å^2^)

**Table:**

	*x*	*y*	*z*	*U*_iso_*/*U*_eq_	Occ. (<1)
C1	1.3321 (2)	0.15359 (17)	0.76907 (6)	0.0129 (2)	
H1	1.4496	0.1155	0.7849	0.015*	0.0329 (9)
C2	1.18845 (19)	0.20920 (16)	0.80593 (5)	0.0109 (2)	
C3	1.0154 (2)	0.26208 (16)	0.78290 (5)	0.0131 (2)	
H3	0.9161	0.2984	0.8080	0.016*	
C4	0.9869 (2)	0.26197 (17)	0.72312 (5)	0.0133 (2)	
H4	0.8686	0.2977	0.7072	0.016*	
C5	1.13375 (19)	0.20895 (16)	0.68709 (5)	0.0108 (2)	
C6	1.30604 (19)	0.15298 (17)	0.70924 (6)	0.0129 (2)	
H6	1.4044	0.1149	0.6841	0.015*	0.9671 (9)
N1	1.21619 (16)	0.20932 (15)	0.86858 (4)	0.01139 (19)	
H1A	1.2127	0.1016	0.8820	0.017*	
H1B	1.1218	0.2704	0.8857	0.017*	
H1C	1.3314	0.2560	0.8771	0.017*	
N2	1.10964 (17)	0.21095 (15)	0.62440 (4)	0.01136 (19)	
H2A	1.2140	0.2587	0.6076	0.017*	
H2B	1.0040	0.2719	0.6151	0.017*	
H2C	1.0959	0.1036	0.6112	0.017*	
O1	1.22585 (15)	−0.22787 (14)	0.49473 (4)	0.01666 (19)	
H1D	1.1180	−0.2666	0.4861	0.025*	
O2	1.44777 (14)	−0.15775 (12)	0.57261 (4)	0.01261 (17)	
O3	1.20902 (15)	−0.39458 (12)	0.58809 (4)	0.01507 (19)	
H3A	1.2990	−0.4584	0.5783	0.023*	
O4	1.09007 (14)	−0.10621 (12)	0.58851 (4)	0.01272 (18)	
O5	1.66727 (16)	0.25284 (15)	0.50298 (4)	0.0190 (2)	
H5	1.7753	0.2093	0.4981	0.029*	
O6	1.64342 (16)	0.10590 (12)	0.60055 (5)	0.01469 (19)	0.9671 (9)
H6A	1.5618	0.0408	0.5861	0.022*	0.9671 (9)
O6B	1.690 (5)	0.096 (4)	0.5735 (15)	0.01469 (19)	0.0329 (9)
H6B	1.5907	0.0361	0.5728	0.022*	0.0329 (9)
O7	1.78483 (14)	0.38754 (12)	0.59744 (4)	0.01286 (18)	
O8	1.43146 (14)	0.34988 (12)	0.57179 (4)	0.01380 (18)	
P1	1.24379 (5)	−0.21374 (4)	0.562311 (13)	0.00959 (7)	
P2	1.63262 (5)	0.28287 (4)	0.569303 (13)	0.00987 (7)	
Cl1	1.54768 (5)	0.08734 (5)	0.796787 (14)	0.01984 (9)	0.9671 (9)
Cl1B	1.5045 (15)	0.0693 (15)	0.6861 (4)	0.01984 (9)	0.0329 (9)

### Atomic displacement parameters (Å^2^)

**Table:**

	*U*^11^	*U*^22^	*U*^33^	*U*^12^	*U*^13^	*U*^23^
C1	0.0096 (5)	0.0148 (5)	0.0141 (5)	0.0021 (5)	−0.0011 (4)	0.0012 (4)
C2	0.0123 (5)	0.0106 (5)	0.0100 (5)	−0.0001 (5)	−0.0002 (4)	0.0011 (4)
C3	0.0119 (6)	0.0155 (5)	0.0119 (5)	0.0031 (5)	0.0008 (4)	0.0000 (4)
C4	0.0108 (6)	0.0171 (6)	0.0120 (5)	0.0040 (5)	0.0002 (4)	0.0008 (4)
C5	0.0133 (6)	0.0101 (5)	0.0091 (4)	−0.0008 (5)	0.0002 (4)	0.0000 (4)
C6	0.0105 (6)	0.0150 (5)	0.0132 (5)	0.0010 (5)	0.0010 (5)	−0.0011 (4)
N1	0.0123 (5)	0.0118 (4)	0.0100 (4)	0.0000 (4)	−0.0011 (4)	0.0004 (3)
N2	0.0123 (5)	0.0127 (4)	0.0091 (4)	−0.0006 (4)	0.0006 (4)	−0.0009 (4)
O1	0.0131 (4)	0.0260 (5)	0.0109 (4)	−0.0043 (4)	0.0005 (3)	−0.0040 (4)
O2	0.0092 (4)	0.0137 (4)	0.0150 (4)	−0.0019 (4)	−0.0006 (4)	0.0000 (3)
O3	0.0132 (5)	0.0101 (4)	0.0220 (4)	−0.0001 (4)	0.0028 (4)	0.0011 (3)
O4	0.0125 (4)	0.0111 (4)	0.0145 (4)	0.0010 (4)	0.0030 (3)	−0.0013 (3)
O5	0.0158 (5)	0.0301 (6)	0.0111 (4)	0.0048 (4)	0.0007 (4)	−0.0045 (3)
O6	0.0168 (5)	0.0097 (4)	0.0176 (5)	−0.0014 (4)	−0.0033 (4)	0.0011 (3)
O6B	0.0168 (5)	0.0097 (4)	0.0176 (5)	−0.0014 (4)	−0.0033 (4)	0.0011 (3)
O7	0.0129 (4)	0.0110 (4)	0.0146 (4)	−0.0013 (4)	−0.0023 (3)	−0.0019 (3)
O8	0.0105 (4)	0.0142 (4)	0.0167 (4)	0.0011 (4)	0.0007 (4)	0.0000 (3)
P1	0.00924 (14)	0.00981 (12)	0.00974 (12)	−0.00071 (12)	0.00054 (11)	−0.00102 (11)
P2	0.00882 (14)	0.00994 (12)	0.01085 (13)	0.00014 (12)	−0.00035 (11)	−0.00110 (11)
Cl1	0.01175 (15)	0.03455 (19)	0.01321 (14)	0.00876 (14)	−0.00169 (12)	0.00062 (13)
Cl1B	0.01175 (15)	0.03455 (19)	0.01321 (14)	0.00876 (14)	−0.00169 (12)	0.00062 (13)

### Geometric parameters (Å, º)

**Table:**

C1—C2	1.3898 (18)	N2—H2B	0.9100
C1—C6	1.3921 (17)	N2—H2C	0.9100
C1—Cl1	1.7228 (14)	O1—P1	1.5678 (10)
C1—H1	0.9500	O1—H1D	0.8400
C2—C3	1.3889 (18)	O2—P1	1.5159 (10)
C2—N1	1.4578 (14)	O3—P1	1.5731 (10)
C3—C4	1.3933 (17)	O3—H3A	0.8400
C3—H3	0.9500	O4—P1	1.5016 (10)
C4—C5	1.3879 (18)	O5—P2	1.5670 (10)
C4—H4	0.9500	O5—H5	0.8400
C5—C6	1.3843 (18)	O6—P2	1.5811 (11)
C5—N2	1.4558 (14)	O6—H6A	0.8400
C6—Cl1B	1.631 (10)	O6—H6B	0.9243
C6—H6	0.9500	O6B—P2	1.54 (3)
N1—H1A	0.9100	O6B—H6B	0.8400
N1—H1B	0.9100	O7—P2	1.5000 (10)
N1—H1C	0.9100	O8—P2	1.5080 (10)
N2—H2A	0.9100		
			
C2—C1—C6	120.83 (12)	C5—N2—H2A	109.5
C2—C1—Cl1	120.36 (10)	C5—N2—H2B	109.5
C6—C1—Cl1	118.81 (10)	H2A—N2—H2B	109.5
C2—C1—H1	119.6	C5—N2—H2C	109.5
C6—C1—H1	119.6	H2A—N2—H2C	109.5
C3—C2—C1	119.64 (11)	H2B—N2—H2C	109.5
C3—C2—N1	119.69 (11)	P1—O1—H1D	109.5
C1—C2—N1	120.65 (11)	P1—O3—H3A	109.5
C2—C3—C4	120.22 (12)	P2—O5—H5	109.5
C2—C3—H3	119.9	P2—O6—H6A	109.5
C4—C3—H3	119.9	P2—O6—H6B	101.4
C5—C4—C3	119.11 (12)	P2—O6B—H6B	109.2
C5—C4—H4	120.4	O4—P1—O2	116.53 (6)
C3—C4—H4	120.4	O4—P1—O1	112.54 (6)
C6—C5—C4	121.54 (11)	O2—P1—O1	104.62 (6)
C6—C5—N2	118.11 (11)	O4—P1—O3	104.82 (5)
C4—C5—N2	120.34 (11)	O2—P1—O3	110.77 (6)
C5—C6—C1	118.63 (12)	O1—P1—O3	107.34 (6)
C5—C6—Cl1B	138.9 (4)	O7—P2—O8	116.93 (6)
C1—C6—Cl1B	102.3 (4)	O7—P2—O6B	108.8 (12)
C5—C6—H6	120.7	O8—P2—O6B	125.5 (13)
C1—C6—H6	120.7	O7—P2—O5	113.36 (6)
C2—N1—H1A	109.5	O8—P2—O5	103.63 (6)
C2—N1—H1B	109.5	O6B—P2—O5	82.8 (13)
H1A—N1—H1B	109.5	O7—P2—O6	105.14 (6)
C2—N1—H1C	109.5	O8—P2—O6	109.93 (6)
H1A—N1—H1C	109.5	O5—P2—O6	107.60 (6)
H1B—N1—H1C	109.5		

### Hydrogen-bond geometry (Å, º)

**Table:**

*D*—H···*A*	*D*—H	H···*A*	*D*···*A*	*D*—H···*A*
N1—H1*A*···O7^i^	0.91	1.76	2.6726 (15)	175
N1—H1*B*···O4^ii^	0.91	1.88	2.7807 (15)	172
N1—H1*C*···O2^iii^	0.91	2.05	2.9155 (15)	158
N2—H2*A*···O8	0.91	1.88	2.7886 (15)	178
N2—H2*B*···O7^iv^	0.91	1.84	2.7450 (15)	178
N2—H2*C*···O4	0.91	1.75	2.6545 (15)	175
O1—H1*D*···O2^v^	0.84	1.90	2.6525 (14)	148
O3—H3*A*···O8^vi^	0.84	1.79	2.5863 (14)	158
O5—H5···O8^vii^	0.84	2.00	2.6585 (14)	134
O6—H6*A*···O2	0.84	1.79	2.5841 (14)	156
O6*B*—H6*B*···O2	0.84	1.84	2.63 (3)	157

Symmetry codes: (i) −*x*+3, *y*−1/2, −*z*+3/2; (ii) −*x*+2, *y*+1/2, −*z*+3/2; (iii) −*x*+3, *y*+1/2, −*z*+3/2; (iv) *x*−1, *y*, *z*; (v) *x*−1/2, −*y*−1/2, −*z*+1; (vi) *x*, *y*−1, *z*; (vii) *x*+1/2, −*y*+1/2, −*z*+1.
